# Recovery from a cycling time trial is enhanced with carbohydrate-protein supplementation vs. isoenergetic carbohydrate supplementation

**DOI:** 10.1186/1550-2783-5-24

**Published:** 2008-12-24

**Authors:** John M Berardi, Eric E Noreen, Peter WR Lemon

**Affiliations:** 1 Precision Nutrition, Inc, Toronto, Ontario, Canada; 2 Department of Health Sciences, Gettysburg College, Gettysburg, Pennsylvania 17325, USA; 3 Exercise Nutrition Research Laboratory, Faculty of Health Sciences, School of Kinesiology, The University of Western Ontario, Ontario N6A 3K7, Canada

## Abstract

**Background:**

In this study we assessed whether a liquid carbohydrate-protein (C+P) supplement (0.8 g/kg C; 0.4 g/kg P) ingested early during recovery from a cycling time trial could enhance a subsequent 60 min effort on the same day vs. an isoenergetic liquid carbohydrate (CHO) supplement (1.2 g/kg).

**Methods:**

Two hours after a standardized breakfast, 15 trained male cyclists completed a time trial in which they cycled as far as they could in 60 min (AM_ex_) using a Computrainer indoor trainer. Following AM_ex_, subjects ingested either C+P, or CHO at 10, 60 and 120 min, followed by a standardized meal at 4 h post exercise. At 6 h post AM_ex _subjects repeated the time trial (PM_ex_).

**Results:**

There was a significant reduction in performance for both groups in PM_ex _versus AM_ex_. However, performance and power decreases between PM_ex _and AM_ex _were significantly greater (p ≤ 0.05) with CHO (-1.05 ± 0.44 km and -16.50 ± 6.74 W) vs C+P (-0.30 ± 0.50 km and -3.86 ± 6.47 W). Fat oxidation estimated from RER values was significantly greater (p ≤ 0.05) in the C+P vs CHO during the PM_ex_, despite a higher average workload in the C+P group.

**Conclusion:**

Under these experimental conditions, liquid C+P ingestion immediately after exercise increases fat oxidation, increases recovery, and improves subsequent same day, 60 min efforts relative to isoenergetic CHO ingestion.

## Background

Several studies have demonstrated that the addition of protein to a carbohydrate recovery beverage can improve short-term glycogen resynthesis vs. carbohydrate supplementation alone [1-4]. However, this area of research is equivocal as a number of other studies have demonstrated similar glycogen resynthesis with carbohydrate + protein or carbohydrate only supplementation [5-10]. As methodological differences may account for these conflicting results, including different types of nutrients, different nutrient timing, different frequency of ingestion, and different exercise modes and intensities, further investigation is needed to clarify which interventions can have the greatest impact on glycogen resynthesis under specific recovery conditions.

Regardless of the conflicting data, it is generally accepted that interventions leading to increased glycogen storage during short-term recovery would likely produce important performance benefits during subsequent high intensity exercise; assuming the exercise bout is of a sufficient duration for glycogen to be limiting. In support of this, Williams et al. [4] and Saunders et al. [11] have demonstrated clear performance benefits during subsequent exercise after the co-ingestion of protein and carbohydrate vs. carbohydrate alone; with the Willams study [4] study suggesting a link between the higher glycogen resynthesis rates and the performance benefits observed.

Previously, we demonstrated that carbohydrate + protein supplements, given early in recovery from a 1 h cycling time trial, enhanced glycogen storage over the following 6 h relative to an isoenergetic carbohydrate condition (1.2 g/kg; [1]). However, when the cycling time trial was repeated (6 h after the initial time trial), we did not observe a significant difference in performance between the carbohydrate + protein and the carbohydrate only conditions. It is possible that the enhanced glycogen synthesis was of insufficient magnitude to enhance performance. Alternatively, it is possible that the subjects' self-selected exercise intensities during the time trial were too low to reveal any benefit of the extra glycogen.

In the present investigation we attempted to determine which of these possibilities was responsible by repeating our experiment. This time we used an exercise test that provided the subjects with more feedback and visual stimulation as well as a virtual opponent. We did this with the hope that the new intervention would increase subject motivation, leading to a higher intensity effort and more closely mimic training and race conditions.

## Methods

### Participants

Fifteen trained cyclists ranging in cycling ability from recreational to elite, as determined by the Ontario Cycling Association, were matched based on measured performance ability and assigned (see details below) to one of two treatment groups (Table 1). Based on a medical history interview, none had neuromuscular, metabolic, or musculoskeletal disorders. In addition, none were using dietary supplements other than multi-vitamins, glucose-electrolye solutions (liquid carbohydrate supplements), or protein supplements. All gave informed written consent according to a protocol approved by the Research Ethics Review Board of the University of Western Ontario.

**Table 1:** Subject physical characteristics, dietary intake, and cycling performance data.

Group	Age (y)	Body Mass (kg)	Baseline Performance (km)	Energy Intake (Kcal/d)	Carb Intake (g/d)	% Carb Intake	Fat Intake (g/d)	% Fat Intake	Protein Intake (g/d)	% Protein Intake
C+P (n = 7)	32.43 ± 8.85	74.76 ± 9.39	14.30 ± 1.64	2974 ± 670	401 ± 123	54%	89 ± 28	28%	128 ± 84	18%
CHO (n = 8)	34.00 ± 9.74	78.98 ± 8.21	14.24 ± 1.64	2628 ± 1307	368 ± 215	55%	87 ± 41	29%	115 ± 62	17%

Subjects were similar (P > 0.05) at baseline.

Values are represented as mean ± SD.

### Experimental protocol

Subjects visited the laboratory on two separate occasions, with at least 48 h between visits, to complete familiarization trials. Subjects also visited the laboratory on a third occasion, at least 7 days after the last familiarization trial, to complete the experimental protocol. The first two visits included 60-min best effort time trials performed at either a 5% or a 7% constant grade using an adjustable fit bicycle placed on a computrainer indoor trainer as outlined below. Whether a participant cycled at 5% or 7% was determined based on self-reported cycling ability, with the stronger cyclists riding a 7% grade, and the weaker subjects riding a 5% grade. This system of handicapping resulted in a similar total distance completed for all cyclists. (For more on this procedure, see Exercise equipment and performance feedback system below).

For these familiarization trials, the object was to cover as great a distance as possible in 60 min. Subjects performed these familiarization trials to get accustomed to the specific performance task they'd be asked to do on the main experimental day. Further, these familiarization trials helped us match subjects according to their best time trial performance. This allowed us to assign them appropriately, according to performance ability, into one of two treatment groups: carbohydrate + protein (C+P; n = 7) or carbohydrate only (CHO; n = 8). In retrospect, this matching technique was successful, resulting in similar distances covered during the experimental day (Am_ex_, see below for details) between the two treatment groups (C+P = 14.3 ± 1.6 km vs. CHO = 14.2 ± 1.6 km, P > 0.05).

On the day prior to the experimental day, the cyclists weighed and recorded all foods and beverages ingested and refrained from exercise. The dietary records were subsequently analyzed using ESHA Food Processor dietary analysis software (ESHA research, Salem, OR).

On the morning of the experiment, subjects consumed a standardized breakfast consisting of cereal (Vector^® ^Meal Replacement, Kellogg Canada Inc), cereal bars (Vector^®^, Kellogg Canada Inc), and skim milk. Thereafter, they completed two questionnaires; a muscle soreness questionnaire [12] and a Profile of Mood States questionnaire [13,14]. Two hours after this meal, body mass was measured using a calibrated weigh scale and a blood sample was taken via indwelling venous catheter. After this, subjects performed a 10-min warm-up, followed by a 60-min all out cycling time trial (AM_ex_), identical in nature to the two familiarization trials each subject had previously completed. Only water (ad libitum) was ingested during the time trial. Expired gases were analyzed during min 10–15 and 40–45 of the time trial, and blood samples were collected immediately pre-, at min 15 and 45, and 5 min post time trial (see Metabolic measurements and blood sampling below).

After completion of the time trial, subjects pedaled lightly for 5-min. Then, (~10-min after the cessation of exercise), each consumed a 1 L post-exercise liquid supplement (CHO or C+P), with a second and third identical drink at 60- and 120-min post-exercise (see Nutritional supplementation below). At 240-min post-exercise (120-min before the second exercise bout) the subjects consumed a meal identical to their breakfast meal. This manipulation ensured that by the end of the 6 h recovery period, both groups had consumed the same amount of energy (21 kcal/kg). During this 6 h recovery period, the subjects consumed only the foods and beverages provided and rested quietly in the laboratory. Six hours after completion of the AM_ex_, the subjects completed a 10-min warm-up which was identical to the warm-up prior to the AM_ex_, followed immediately by another 60-min time trial (PM_ex_). All procedures were identical to the AM_ex_.

### Exercise equipment and performance feedback system

A fully adjustable Serotta Size Cycle (Saratoga Springs, NY) with a 12 gear internal hub rear wheel and a Computrainer^® ^Pro laboratory/research edition (Seattle, WA) indoor trainer was used for all time trials. The Computrainer^® ^Pro was interfaced to a laboratory computer and monitor allowing for real-time visual feedback of each performance. This software package essentially presents the subjects with a computer game in which there is a virtual cyclist whose speed is controlled by the subject's actual efforts on the bicycle, and a virtual opponent (to serve as a pacer) that represents either 1) a recording of a past performance, or 2) is set to mimic the subject's own speed. In addition to the computer characters and race course, this program also displays cycle speed, distance travelled, subject position relative to the computer-generated opponent, work output (current watts and average watts), heart rate (heart rate monitor attached to an ear lobe) revolutions per minute, and energy expenditure. These data were calculated using mathematical models included in the Computrainer^® ^Pro software package (Computrainer^® ^Pro 3D Graphics Package). The models used take into account variables such as the cyclist's mass as well as the percent grade of the course and determines how fast the computer cyclist is going based on how many watts the subject is able to generate. The end result is a virtual replication of an outdoor ride with the advantage of having a virtual opponent.

As mentioned above, subjects were assigned to one of two different courses, either a 5% or a 7% grade depending the cyclist's performance ability. Similar to an actual road course, riding up a hill with a greater slope will result in a slower speed for a given work output. We chose this approach to reduce the variability of the performance measure. However, it's important to note that the exertional efforts of the subjects, regardless of the percent grade they cycled at, were not impacted since percent grade does not impact the absolute amount of work performed, just the net distance travelled. For example, if a subject was able to average 250 w for an hour time trial, he would average the same workload regardless of whether he rode a course with a 5% or a 7% grade, the only difference is that he would travel a greater distance on the course with the 5% grade. Regardless, we matched subjects based on the percent grade they cycled at. In the C+P condition (n = 7), 2 participants cycled at 5% grade and 5 participants cycled at 7% grade. In the CHO condition (n = 8), 3 participants cycled at 5% grade and 5 participants cycled at 7% grade.

Prior to each time trial, the rear wheel was inflated to 120 psi, and both the tire and the roller on the Computrainer^® ^were cleaned with alcohol. Immediately following the 10 min warm up, and prior to all testing, the rear wheel resistance of the Computrainer^® ^was calibrated according to the manufacturers instructions. For the initial test for each subject, the rear wheel press on force was set between 2.00–2.5 lb. This value was recorded and for all subsequent tests the final calibrated press on force was within 0.2 lb of this initial value. Immediately after calibration, the subjects cycled as far as they could in 60-min treating the exercise bout as a time trial. A fan was directed on them to facilitate cooling. During the AM_ex_, subjects consumed water ad libitum and the amount ingested was recorded for use during the PM bout (mean water intake during exercise was 1.25 ± 0.40 L).

In order to prevent bias, subjects were instructed to do their very best prior to each trial but no verbal encouragement was provided during any of the exercise bouts. For the initial familiarization trial, the computer pacer was set using a 2 sec delay. This meant that the pacer based its speed on how fast the subject was going, but did not respond immediately to changes in the subject's speed. Subjects, however, were told that the pacer's speed was chosen based on their individual abilities and were encouraged to try and put as much distance between themselves and the pacer as possible. This strategy changed with the second familiarization trial. In this trial, the subject's performance on the first familiarization trial was used as the pacer. Finally, the best performance of the two familiarization trials was then used as the pacer during the AM_ex _trial, while the AM_ex _performance was used as the pacer during the PM_ex _trial. All performances were recorded and prominently displayed on a chalkboard in the laboratory, with the hopes that this would foster a friendly competition between the subjects and further motivate them to give a maximal effort for each trial

### Metabolic measurements and blood sampling

During min 5–15 and 35–45 of the AM and PM_ex_, expired gases were collected breath-by-breath using a Sensormedics Vmax 29 metabolic cart system (VIASYS Healthcare, Yorba Linda, CA); data from minutes 10–15 and 40–45 were then analyzed. Prior to each test, both gas concentration and flow were calibrated according to manufacturer's specifications. Breath by breath data were averaged for the 5-min measurement interval and values for VO_2 _and RER were calculated. Using these data, the oxidation rate (gmin^-1^) for carbohydrate and fat were calculated during minutes 10–15 and 40–45 of AM (AM15 and AM45) and PM_ex _(PM15 and PM45) according to Rowlands and Hopkins [15]:

Carbohydrate Oxidation = 4.5850 • VCO_2 _- 3.2255 • VO_2_

Fat Oxidation = 1.6946 • VCO_2 _- 1.7012 • VO_2_

No correction was made for protein oxidation.

Immediately before and after AM and PM_ex_, as well as at min 15 and 45 of both AM_ex _and PM_ex _time trials, blood was collected via an indwelling venous catheter into evacuated glass tubes. PRE and POST_ex _samples were collected in serum separator tubes while 15- and 45-min samples were collected in tubes containing a glycolytic inhibitor and all tubes were immediately placed on ice. This blood was separated and the serum frozen at -150 degrees C for later analysis of glucose and lactate (YSI 2300 Stat Plus Glucose and Lactate Analyzer, YSI Life Sciences, Yellow Springs, OH).

### Questionnaires

Prior to AM and PM_ex_, subjects completed a Profile of Mood States (POMS) questionnaire and a muscle soreness scale. The POMS identifies and assesses transient, fluctuating affective mood states including Tension-Anxiety, Depression-Dejection, Anger-Hostility, Vigor-Activity, Fatigue-Inertia, and Confusion-Bewilderment [14]. This 65-question survey has been shown to be particularly useful in measuring change in mood states over time, especially in athletic populations [13] as it presents subjects with a number of descriptors such as "tired", "energetic", etc. and inquires as to their present mood and whether they feel this way: a) not at all, b) a little, c) moderately, d) quite a bit, and e) extremely. Each response is given a numeric value and contributes to one of the aforementioned mood domains.

The muscle soreness scale is a 10-point visual analogue scale, 10 cm in length, with each point exactly 1 cm from the last [12]. These data represent ratio data with a true zero (0 – no soreness, 10 – extreme soreness) designed to give an indication of changing muscle soreness over time. It's important to note that with the short time interval between AM and PM_ex _in this investigation, this soreness scale doesn't necessarily represent Delayed-Onset Muscle Soreness, which is typically associated with a longer lag time between exercise and onset. Rather, it simply offers a global assessment of self-reported soreness/localized fatigue.

### Nutritional supplementation

The standardized breakfast (consumed 120-min prior to AM_ex_) consisted of cereal (Vector^® ^Meal Replacement, Kellogg Canada Inc), cereal bars (Vector^®^, Kellogg Canada Inc), and skim milk (7 kcal/kg; 1.2 g/kg C, 0.3 g/kg P, 0.1 g/kg F). The liquid supplements ingested 10-, 60-, and 120-min post-exercise were CHO + protein (C+P; 33% maltodextrin, 33% glucose, and 33% whey protein hydrolysates) and CHO (100% maltodextrin). Each supplement plus 3 g of Crystal Light^® ^(to provide color and flavor consistency between drinks) was dissolved in 1 L of water, ensuring an 8–12% (by mass) solution. Drink energy content was 4.8 kcal/kg body mass per drink (C+P [0.8 g/kg carbohydrate and 0.4 g/kg protein]; CHO [1.2 g/kg carbohydrate]). The solid meal ingested 4 h post-AM_ex _was identical to the standardized breakfast for both C+P and CHO (7 kcal/kg). These nutritional interventions ensured that by the end of the 6 h recovery period, both groups had consumed the same amount of dietary energy (21.4 kcal/kg), with a varying macronutrient intake. The C+P group ingested a total of 3.6 g/kg carbohydrate, 1.5 g/kg protein, and 0.1 g/kg fat during the 6 h recovery period, and the CHO group ingested 4.8 g/kg carbohydrate, 0.3 g/kg protein, and 0.1 g/kg fat. Total energy intake in both groups was 1553 kcal during the 6 h recovery period with C+P ingesting 266 g carbohydrate, 111 g protein, 7 g fat, and the CHO ingesting 355 g carbohydrate, 22 g protein, 7 g fat. Estimated total energy expenditure was ~2200 – 2500 kcal (exercise expenditure plus resting metabolic rate) and energy balance during the entire study period was controlled by providing a mean energy intake of 2100 kcal (breakfast plus recovery nutrition).

### Statistical analysis

Statistical analyses were performed using SPSS software (SPSS, Version 10, Chicago, IL). Values are reported as means ± SEM. A group × time ANOVA with repeated measures was used to determine differences between distance covered, mean watts production, heart rate, body mass, muscle soreness, mood scores, serum glucose, serum lactate, estimated carbohydrate oxidation, estimated fat oxidation, and estimated energy utilization. Planned comparisons were also conducted between AM15 and AM45 as well as PM15 and PM45 (2 × 2 ANOVA) to examine group differences in blood glucose and blood lactate during AM_ex _and PM_ex_. In all analyses, when indicated, a Tukey's Honestly Significant Difference post-hoc was used. Power calculations were performed on the main criterion measures (mean distance traveled and mean power output). In addition, a t-test was used to determine differences in nutritional intake between groups on the day prior to testing. For all tests, statistical significance was set at p ≤ 0.05. Finally in the reported results below, when no group differences were seen between CHO and C+P, group means were collapsed into single AM_ex _and PM_ex _values.

## Results

### Exercise performance

Mean distances travelled for the same-day time trial performances were as follows:

14.3 ± 1.64 km during AM_ex _and 14.0 ± 1.57 km during PM_ex _in C+P

14.24 ± 1.64 km during AM_ex _and 13.19 ± 1.70 km during PM_ex _in CHO

Mean power output for the same-day time trial performances were as follows:

210.43 ± 57.57 W during AM_ex _and 206.57 ± 54.52 W during PM_ex _in C+P

215.63 ± 56.31 W during AM_ex _and 199.13 ± 56.87 W during PM_ex _in CHO

**Figure 1:**
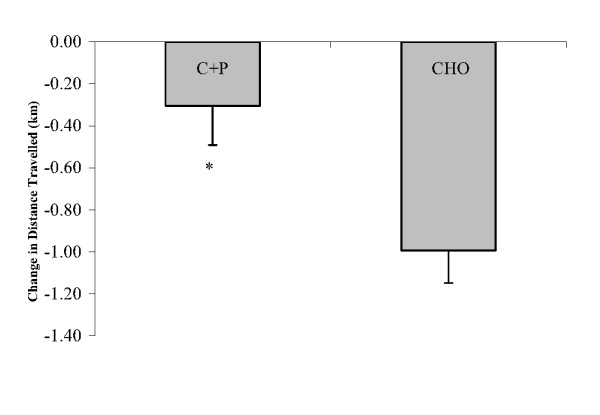
**Decrease in total distance traveled in a time trial (PM_ex_) performed 6 h after an initial time trial (AM_ex_)**. During the recovery period between AM_ex _and PM_ex _nutritional interventions included early post exercise carbohydrate + protein supplements (C+P) and a later solid meal and early carbohydrate supplement (CHO) and a later solid meal. *The performance decrement in C+P is significantly less than in CHO (p ≤ 0.05).

**Figure 2:**
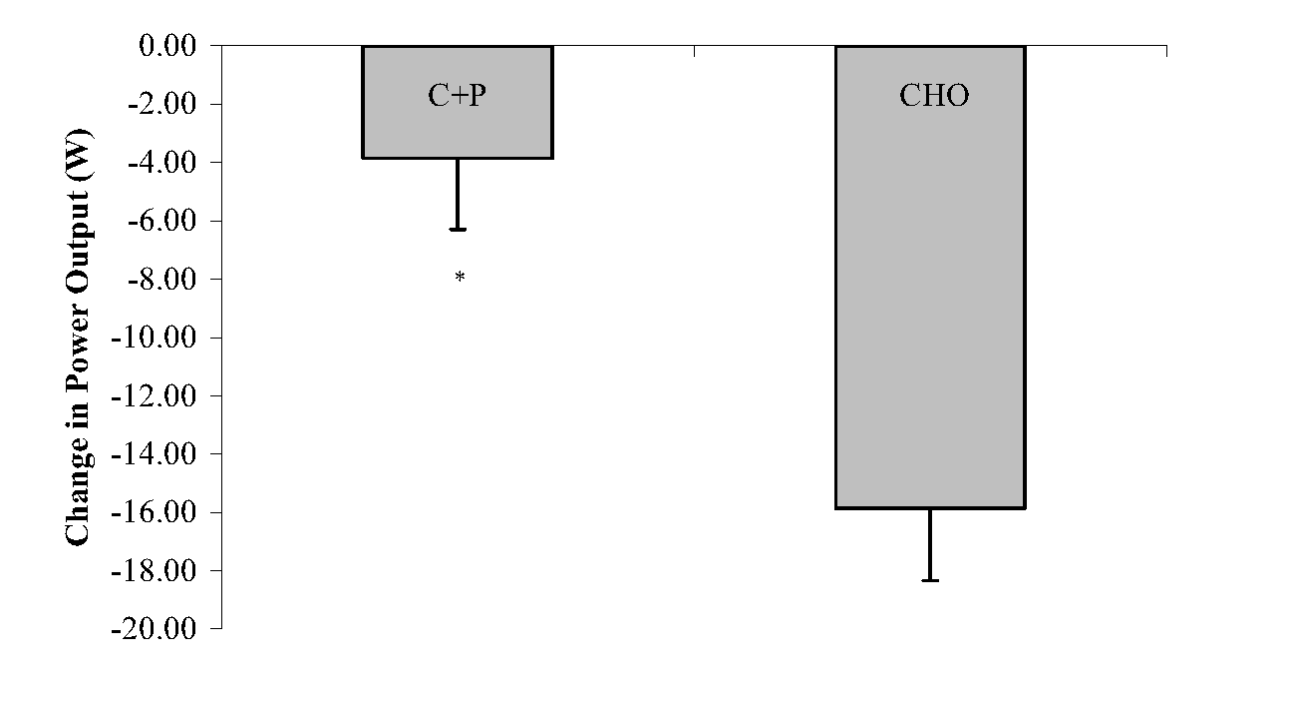
**Decrease in power output during a time trial (PM_ex_) performed 6 h after an initial time trial (AM_ex_)**. During the recovery period between AM_ex _and PM_ex _nutritional interventions included early post exercise carbohydrate + protein supplements (C+P) and a later solid meal and early carbohydrate supplement (CHO) and a later solid meal. *The decrease in power output in C+P is significantly less than in CHO (p ≤ 0.05).

Both groups performed significantly better in the AM_ex _performance compared to the PM_ex _performance, with no significant differences observed between the groups for the AM_ex _ride. However, the reduction in distance traveled and power output during PM_ex _(vs AM_ex_) was significantly less in the C+P condition (-0.30 ± 0.19 km and -3.86 ± 2.44 W) relative to CHO (-1.05 ± 0.16 km and -16.50 ± 2.39 W; Fig 1 &2). Statistical power calculations indicate power factors of 0.835 for distance traveled and 0.965 for mean power output.

### Muscle soreness

There was a significant time effect observed when comparing PM_ex _(a rating of 3.14 ± 0.51; 10 point scale) vs AM_ex _(a rating of 1.57 ± 0.48) with no significant group differences or group by time interactions.

### Profile of mood states

There was a significant time effect for both groups in the Fatigue-Inertia domain, with all subjects feeling more fatigued prior to PM_ex _relative to AM_ex_. However subjects in C+P reported significantly smaller increases in Fatigue-Inertia (+3.29 ± 0.47) vs CHO (+8.57 ± 2.29) as seen below.

Fatigue-Inertia from AM_ex _to PM_ex _(CHO):

3.71 ± 1.52 to 12.29 ± 2.79

Fatigue-Inertia from AM_ex _to PM_ex _(C+P):

4.43 ± 1.54 to 7.71 ± 1.74

There was also a significant time effect in the Vigor-Activity domain with all subjects, regardless of group, reporting higher values prior to AM_ex _vs PM_ex_.

Vigor-Activity from AM_ex _to PM_ex_

19.10 ± 2.19 to 13.38 ± 3.08

However, there were no group, time, or group by time interactions between AM_ex _and PM_ex _for scores in any other domain.

Tension-Anxiety from AM_ex _to PM_ex_

3.57 ± 1.74 to 3.00 ± 1.83

Depression-Dejection from AM_ex _to PM_ex_

2.00 ± 1.02 to 2.62 ± 1.43

Anger-Hostility from AM_ex _to PM_ex_

2.71 ± 1.09 to 3.05 ± 1.54

Confusion-Bewilderment from AM_ex _to PM_ex_

0.67 ± 1.12 to 1.67 ± 1.46

### Heart rate

There were no significant differences between groups in heart rate between AM_ex _(168.57 ± 3.45 bpm) and PM_ex _(167.71 ± 2.73 bpm).

### Body mass

Body mass prior to AM_ex _(77.75 ± 1.64 kg) and PM_ex_(77.72 ± 1.66 kg) was similar (p > 0.05) with no difference between groups.

### Serum glucose and lactate

**Figure 3:**
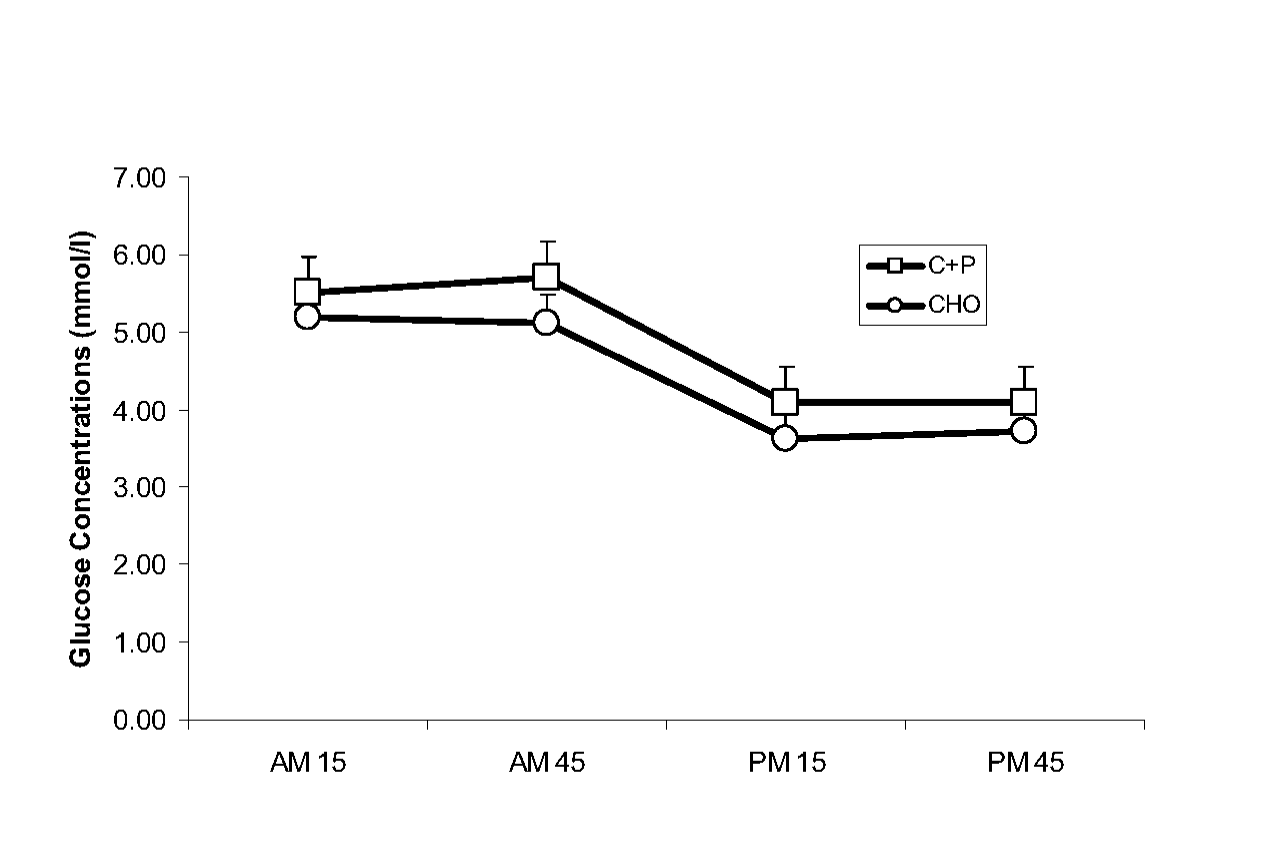
**Serum glucose concentration during AM_ex _and PM_ex_**. During the recovery period between AM_ex _and PM_ex _nutritional interventions included early post exercise carbohydrate + protein supplements (C+P) and a later solid meal and early carbohydrate supplement (CHO) and a later solid meal. Serum glucose concentration decreased from AM45 to PM15 (p ≤ 0.05) but there were no other differences over time or between conditions.

**Figure 4:**
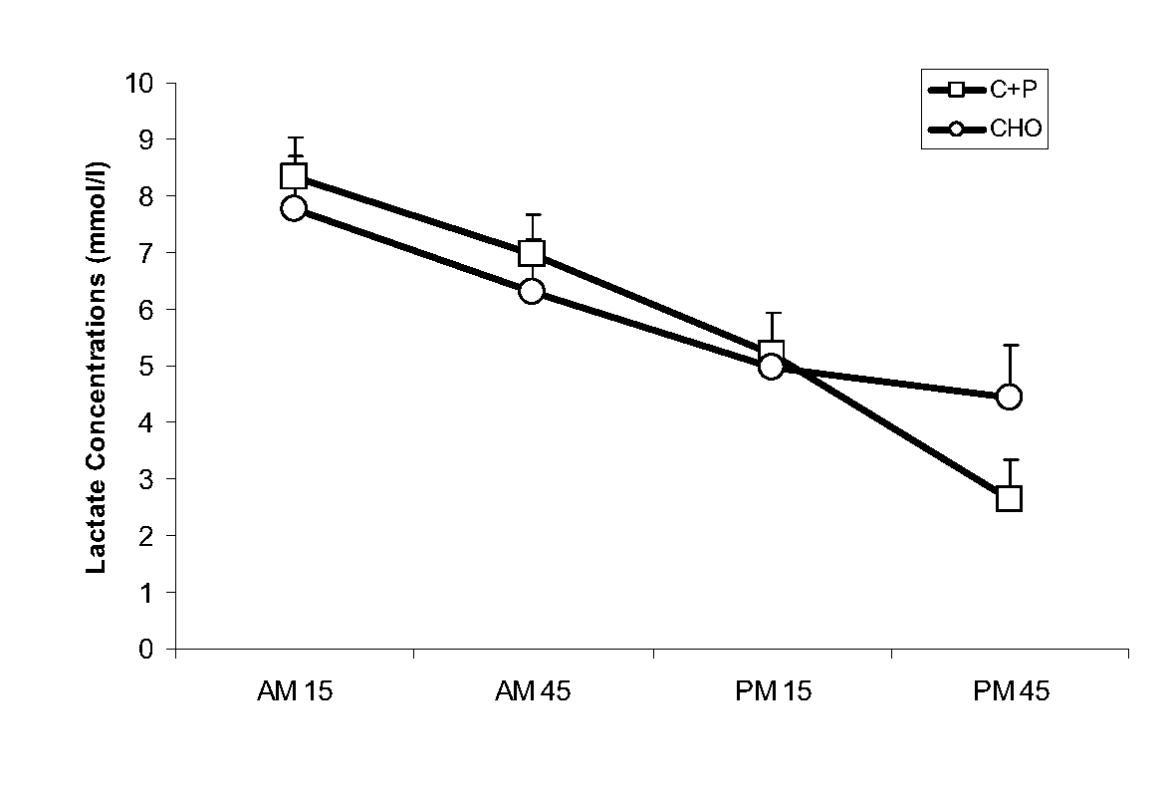
**Serum lactate concentration during AM_ex _and PM_ex_**. During the recovery period between AM_ex _and PM_ex _nutritional interventions included early post exercise carbohydrate + protein supplements (C+P) and a later solid meal and early carbohydrate supplement (CHO) and a later solid meal. Serum lactate concentration decreased at each time point from AM15 to PM15 (p ≤ 0.05) but there were no other differences over time or between conditions.

There was a significant time effect with serum glucose concentration decreasing from AM45 to PM15 in both groups (Fig 3). Similarly, there was a significant time effect for serum lactate, with both groups decreasing from AM15 to PM 15 (Fig 4). Planned comparisons of AM15 vs AM45 and PM15 vs PM45 (2 × 2 ANOVA) revealed no group, time or interaction effects.

### Carbohydrate and fat oxidation

**Figure 5:**
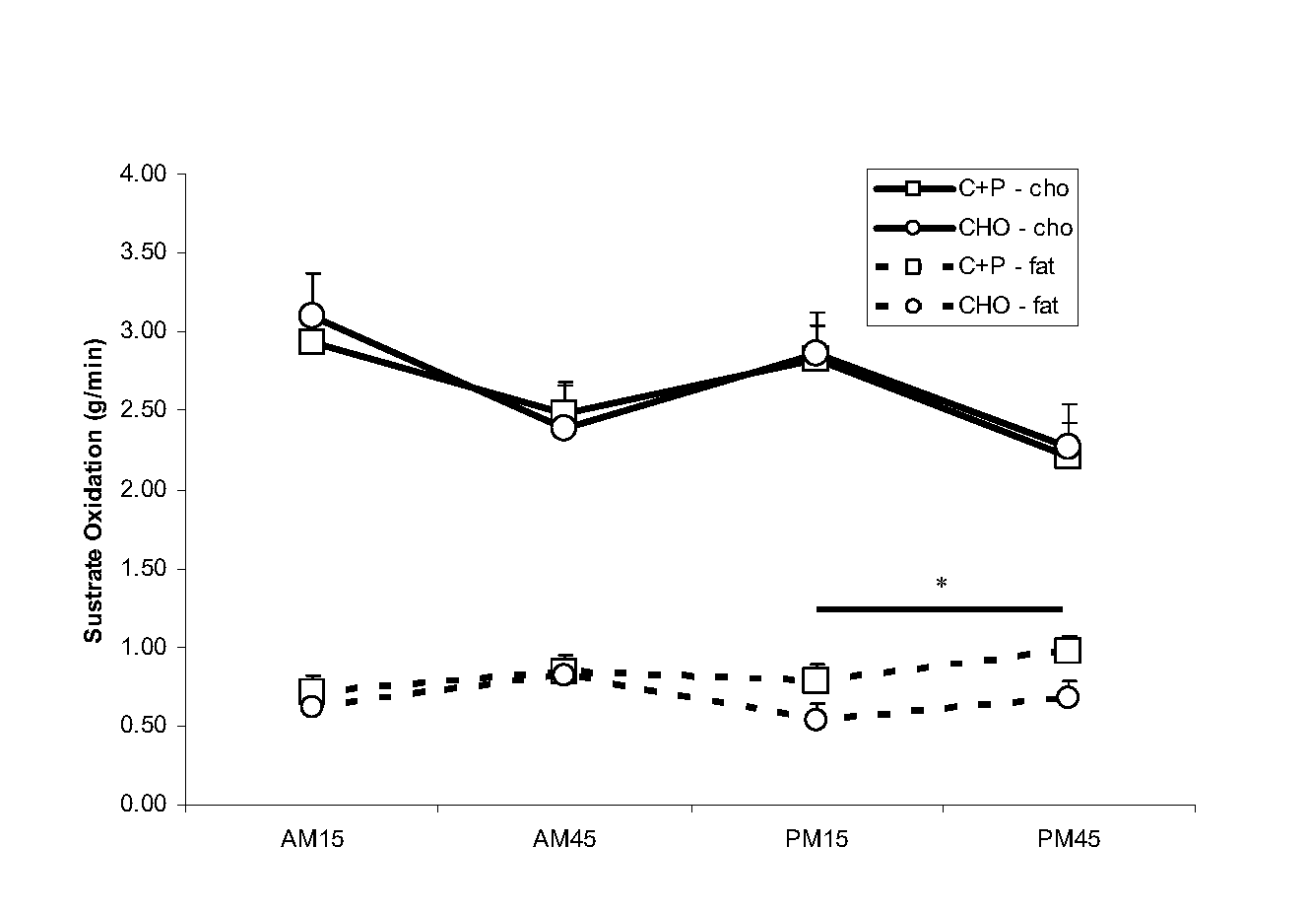
**Carbohydrate and fat oxidation during AM_ex _and PM_ex_**. During the recovery period between AM_ex _and PM_ex _nutritional interventions included early post exercise carbohydrate + protein supplements (C+P) and a later solid meal and early carbohydrate supplement (CHO) and a later solid meal. There was a time effect for carbohydrate oxidation but no group effects or group by time interactions. *While rates of fat oxidation were higher in C+P during both PM time points (vs. AM_ex_); in CHO, rates of fat oxidation were lower during both PM time points relative to the AM time points (p ≤ 0.05).

There was a significant time effect observed for estimated carbohydrate oxidation (Figure 5) with greater rates at AM15 vs AM45, at PM15 vs AM45, and at PM15 vs PM45. No group or group by time interactions were observed.

In both conditions, rates of fat oxidation increased significantly during both AM_ex _(from 0.72 ± 0.04 gmin^-1 ^to 0.89 ± 0.07 gmin^-1^) and PM_ex _(from 0.76 ± 0.04 gmin^-1 ^to 0.89 ± 0.06 gmin^-1^; Fig 5). In addition, while rates of fat oxidation were significantly greater at PM15 vs AM15 in C+P (0.79 ± 0.05 gmin^-1 ^vs 0.71 ± 0.07), the rate of fat oxidation was significantly reduced in CHO at PM15 vs AM15 (0.54 ± 0.04 gmin^-1 ^vs 0.61 ± 0.04). Also, fat oxidation was significantly greater in C+P at PM45 vs AM45 (0.97 ± 0.09 gmin^-1 ^vs 0.85 ± 0.07 gmin^-1^), but it was significantly reduced (PM45 = 0.68 ± 0.05 gmin^-1 ^vs AM45 = 0.82 ± 0.06) in the CHO. Finally, fat oxidation rates were significantly greater in C+P at PM15 (0.79 ± 0.05 gmin^-1^) and PM45 (0.97 ± 0.09 gmin^-1^) vs CHO at PM15 (0.54 ± 0.04 gmin^-1^) and PM45 (0.68 ± 0.05 gmin^-1^).

## Discussion

The main finding of this study is that liquid C+P supplements given early during a 6 h post-exercise recovery period helped subjects better maintain subsequent time trial performance and power output relative to isoenergetic liquid CHO supplements given early during recovery. Although both conditions performed significantly worse in the PM_ex _versus the AM_ex_, the 3.86 W decrease in the PM_ex _observed with C+P was significantly less than the 16.50 W decrease observed with CHO in the PM_ex_. This corresponded to a 0.30 km reduction in distance traveled in the PM_ex _versus the AM_ex _with C+P and a 1.05 km reduction with CHO (p ≤ 0.05).

Previously, using an identical feeding protocol, we measured a greater (p ≤ 0.05) muscle glycogen storage between exercise sessions in the C+P condition vs CHO condition [1]. Assuming a similar response to C+P feedings in these subjects, which is reasonable, the additional glycogen could, in part, explain these findings.

The performance data observed in the current study are consistent with several other studies that have found an improvement in subsequent exercise performance when C+P is taken during the recovery period [4,11,12]. However, not every study has found an improvement in subsequent exercise performance following ingestion of a carbohydrate + protein recovery drink. Karp et al found that both chocolate milk and a commercial glucose electrolyte drink consumed in the recovery period resulted in an increase in subsequent exercise performance compared to a protein + carbohydrate recovery drink [16]. It is unclear why chocolate milk, which contains protein and carbohydrate, was better than a protein + carbohydrate drink, but the authors speculated that differing carbohydrate composition may have played a role.

Interestingly, previous research from our group, using a very similar protocol to the present study, also failed to show an improvement in subsequent exercise when a protein + carbohydrate recovery drink was ingested versus carbohydrate only [1]. The fact that there was no improvement in the protein + carbohydrate versus the carbohydrate only trials is surprising since glycogen resynthesis was 22% greater with carbohydrate + protein recovery drinks (p ≤ 0.05) and it is generally accepted that initial muscle glycogen levels are a significant predictor of prolonged endurance performance [17,18]. While it is certainly possible that the increased muscle glycogen content seen in the carbohydrate + protein condition was still of insufficient magnitude to impact on subsequent exercise performance, it is also possible that the exercise intensity wasn't high enough where muscle glycogen levels are a factor for exercise of this duration. Muscle glycogen levels are critical for sustaining higher intensity exercise [17,19] but at, or below, about 70% of max, exercise can be continued with depleted muscle glycogen stores as long as plasma glucose levels are maintained [20].

Taken with the results of this investigation, we speculate that the difference between this investigation and our previous work is a function of the different exercise modalities selected. In our previous work [1] we used a wind trainer device and, although the wind trainer device recorded total distance traveled, the cyclists did not receive continuous feedback about heart rate, watt production, speed, or distance traveled relative to prior bouts. As a result, subject motivation was likely lower in the previous investigation. In this study, however, these variables, along with a virtual competitor, were presented. In response, subject motivation was likely higher due to their ability to "race against" their previous performances (in both familiarization testing and during the actual experimental trials). Indeed, to support this notion, estimated exercise intensity (based on HR data) over the 1-h exercise bout was greater in the present study (~76–80% VO2max) vs the previous study (~70–73% VO2max). Therefore, subjects in the previous study may not have been motivated enough (and therefore did not cycle intensely enough during the time trial) for the supplement intervention to demonstrate benefit. Indeed, motivated elite-level cyclists typically perform typical time trial bouts at greater intensities than 70 – 73% VO2max. Future investigations, as well as comparisons between individual studies, should therefore consider carefully subject motivation and the resulting exercise intensity self-selected when investigating the impact of macronutrient composition on actual race performance and/or simulated race performance.

Consistent with the improvements seen in performance recovery in the present study, analysis of the POMS data reveal that subjects ingesting C+P felt less fatigued (p ≤ 0.05) at the start of PM_ex _when compared to CHO. Mean fatigue scores prior to AM and PM_ex _were 4.4 ± 1.5 and 7.7 ± 1.7 (+3.3 ± 0.5) for C+P vs 3.7 ± 1.5 and 12.3 ± 2.8 (+8.6 ± 2.3) for CHO. While we're unsure as to whether there is a direct link between this perception of fatigue and performance, future studies might explore this relationship to determine whether the benefit of C+P supplementation is psychological, physiological, or some combination of the two.

Previously, Rowlands et al. [15] have shown pre-exercise meals providing a high fat (28 g protein, 15 g carbohydrate, 102 g fat) or a high protein content (83 g protein, 122 g carbohydrate, 36 g fat) led to increased fat oxidation during exercise of varying intensities (from 55% to 82% of VO_2 _max) relative to high carbohydrate meals (28 g protein, 258 g carbohydrate, 6 g fat). As insulin is a potent inhibitor of fat mobilization and lipolysis [21,22] greater circulating insulin may have reduced fat oxidation with CHO in both the Rowlands study [15] and in the present study. However, this may not be the whole explanation as insulin concentration was also greater in the protein condition in the Rowlands study [15], yet fat oxidation was not reduced.

It is well known that protein ingestion also increases plasma glucagon concentration [23,24]. Further, glucagon-stimulated lipolysis, which may occur primarily in the liver, has been shown to increase rates of fat oxidation even with concomitant increases in plasma insulin concentration [25]. Indeed, in the study by Rowlands et al. [15] plasma glucagon and fat oxidation rates were both highest with the high protein meal.

As a result of these data, and research by Forslund et al. [26], in which increased 24 h fat oxidation rates were demonstrated when subjects ingested a high protein (2.5 gkg^-1^) vs a normal protein diet (1.0 gkg^-1^), the increased fat oxidation seen in C+P in this investigation may have been the result of a higher protein intake. We did not, however, directly measure plasma insulin and glucagon in this study, so this conclusion remains speculative. Further, as we did not correct metabolic data for protein oxidation, comparisons between carbohydrate oxidation and fat oxidation from our study and previous work (in which protein oxidation has been accounted for) will necessarily be limited.

## Conclusion

In conclusion, under the present study conditions, liquid C+P supplements ingested early during recovery better maintain subsequent same day best effort time trial performance vs. isoenergetic liquid CHO supplements given early during recovery. Decrements in both distance traveled and mean power output during PM_ex _were significantly less with C+P vs CHO. Furthermore, subjective fatigue scores were lower prior to PM_ex _with C+P vs CHO. Finally, increases in fat oxidation were observed in the C+P condition vs. CHO. These findings may be important considering that most endurance athletes concern themselves primarily with carbohydrate intake and often fail to recognize the potential benefits of protein with respect to performance recovery.

## Competing interests

The authors declare that they have no competing interests.

## Authors' contributions

JMB participated at the lead author and was responsible for the study design, screening and recruitment, data collection, data analysis and interpretation, and for the final draft of the manuscript. EEN also assisted in the study design, screening and recruitment, data collection, data analysis and interpretation, and for the final draft of the manuscript. PWR, acting as a thesis advisor and senior author, was responsible for assisting in the study design, data analysis and interpretation, and final draft. All of the authors have read and approved the final draft.
